# Congenital intestinal malrotation in adolescent and adult patients: a 12-year clinical and radiological survey

**DOI:** 10.1186/s40064-016-1842-0

**Published:** 2016-03-01

**Authors:** Britt Husberg, Karin Salehi, Trevor Peters, Ulf Gunnarsson, Margareta Michanek, Agneta Nordenskjöld, Karin Strigård

**Affiliations:** Department of Clinical Sciences, Danderyd Hospital, Stockholm, Sweden; Department of Surgery, Ersta Hospital, Stockholm, Sweden; Department of Clinical Intervention and Technology, CLINTEC, Karolinska Institutet, Stockholm, Sweden; Department of Gastrointestinal Surgery, Karolinska University Hospital, Stockholm, Sweden; Department of Women’s and Children’s Health, and Center for Molecular Medicine, Karolinska Institutet, Stockholm, Sweden; Unit of Paediatric Surgery, Astrid Lindgren Children Hospital, Stockholm, Sweden; Department of Radiology, Karolinska University Hospital, Stockholm, Sweden; Department of Surgical and Perioperative Sciences, Umeå University Hospital, 901 87 Umeå, Sweden

**Keywords:** Malrotation, Intestinal volvulus, Adult, Ladd’s procedure

## Abstract

Congenital intestinal malrotation is mainly detected in childhood and caused by incomplete rotation and fixation of the intestines providing the prerequisites for life-threatening volvulus of the midgut. The objective of this study was to evaluate a large cohort of adult patients with intestinal malrotation. Thirty-nine patients, 15–67 years, were diagnosed and admitted to a university setting with congenital intestinal malrotation 2002–2013. The patients were divided into three age groups for stratified evaluation. Medical charts were scrutinized, and clinical outcome of surgery was reviewed. Twelve patients presented as emergency cases, whereas 27 were admitted as elective cases. Diagnosis was established in 33 patients who underwent radiological investigation and in the remaining 6 during surgery. A Ladd’s operation was performed in 31 symptomatic patients; a conservative strategy was chosen in eight cases. Volvulus was more common in the younger age group. Twenty-six surgically treated patients were available for telephone interview, 1–12 years after surgery. All patients, except one, regarded their general condition improved to a high degree (n = 18) or with some reservation (n = 7). Twelve patients suffered remaining abdominal pain of a chronic and diffuse character. Due to recurrence of malrotation six patients were reoperated. Symptomatic malrotation occurs in both children and the adult population. Improved awareness and an accurately performed CT scan can reveal the malformation and enable surgical treatment. A Ladd’s procedure relieved most patients from their severe complaints even when a history of several years of suffering existed.

## Background

In congenital intestinal malrotation an impaired embryological development of the gut causes incomplete rotation and fixation of the intestines to the abdominal wall (Dott 1923). The fulfillment of the third embryonic rotation includes the traversing of the duodenum to the left side of the abdomen, forming the ligaments of Treitz, and the migration of the ileo-caecal junction to the lower right abdominal quadrant. The fixation of the full-length bowel is complete during the twelfth week (Penco et al. 2007). Congenital malformations such as diaphragmatic hernia, omphalocele or gastroschisis are associated with a similar but secondary incomplete rotation and fixation of the intestines (Torres and Ziegler 1993).

The inadequate fixation of the bowel alongside remaining embryonic fibrous adhesions, the Ladd’s bands (Ladd 1932, 1936), may give rise to a variety of intestinal malfunction. In the worst case scenario, malrotation may develop into a midgut volvulus with torsion causing high risk of ischemia and necrosis of the parts of the intestine supplied by the superior mesenteric artery. This life-threatening condition is well known among pediatric surgeons and is always considered when physicians treat critically ill infants with abdominal symptoms and unknown diagnoses.

Malrotation has primarily been diagnosed in early childhood, with estimated onset of symptoms during the first year of life in 90 % of the cases (Vaos and Misiakos 2010; Pickhardt and Bhalla 2002; Stewart et al. 1976). There are recent reports of manifestation later in life, both as emergency conditions or more chronic gastrointestinal symptoms (Penco et al. 2007; Pickhardt and Bhalla 2002; Nehra and Goldstein 2011). The exact incidence of intestinal malrotation is thus still difficult to determine. It was earlier described to be approximately 0.2 % (Stewart et al. 1976; Donnellan and Kimura 1996; Clark and Oldham 2002), but an incidence up to 1 % has been reported (Adams and Stanton 2014). Improved radiological facilities, including multi-detector CT-scans, provide new possibilities to identify anatomical aberrations.

During a 12-year period, we have treated 39 consecutive cases of adult malrotation at the Karolinska University Hospital, Huddinge. The aim of this study was to increase knowledge concerning this diagnosis by describing symptoms, treatment and clinical outcome in our cohort of adolescent and adult patients with intestinal malrotation.

## Methods

### Patients

Thirty-nine patients, 22 females and 17 males, aged between 15 and 67 years, were diagnosed with congenital intestinal malrotation. The patients were prospectively investigated at the Karolinska University Hospital from 2002 to 2013. After identification of the first patient, it was decided to prospectively monitor patients treated for malrotation in order to analyze and publish data when a reasonable number of patients had been treated.

### Medical charts

All medical records were evaluated with regards to symptoms, surgical procedures, previous disorders and outcomes. For analysis of differences according to age, the patients were divided into three groups (15–20, 21–50, 51–67 years).

### Radiological diagnostics

To establish the degree of malrotation, the radiologist identified the position of the duodenum and the proximal small bowel, the location of the caecum and the orientation of the mesenteric vessels using intravenous, per oral as well as intrarectal contrast (triple-contrast). Twisting of the mesentery of the small bowel, the “whirlpool-sign”, typical for a volvulus was noted. This evaluation was also re-scrutinized and confirmed independently by one dedicated radiologist.

### Surgery

Symptomatic malrotation was treated by corrective surgery according to the technique originally described by Ladd. If a volvulus was present, the intestines were de-rotated in a counter clockwise manner and all Ladd’s bands were carefully removed and dissected. If needed, the mesentery was broadened and the adhesions surrounding the mesenteric vessels dissected in order to avoid future recurrence of volvulus. When the dissection was done, the small bowel was placed to the right and the colon to the left side of the abdominal cavity in a “non-rotational” position. Two different surgeons registered data from medical charts on these surgical details independently.

### Follow up

The patients were routinely assessed 6 weeks, 6 months and 12 months after surgery. After that, occasional contact occurred if further complaints presented. During 2012–2013 a research nurse performed telephone interviews with a semi-structured concept concerning the patients’ past and present situation and possible remaining symptoms after surgery. The questions focused on remaining intense or chronic pain, postprandial nausea, vomiting and constipation. Patients were also asked whether they regarded their general physical condition as improved to a high degree, improved with some reservation or without any notable improvement.

### Ethical considerations

The Regional Ethical Review Board approved this study 12-06-20. Dnr 2012/957-31/3.

## Results

### Clinical data

Twelve patients presented as emergency cases, whereas the remaining 27 were admitted on an elective basis. The most common symptom was abdominal pain, followed by signs of intestinal obstruction (Table 1). Another predominant symptom was sensations of extreme fullness and discomfort after meals, sometimes followed by nausea and vomiting, described by 29 patients (Table 2). Thirteen of these patients were previously assessed and diagnosed with gastro-oesophageal reflux. In six cases the diagnosis was achieved during surgical treatment focused on other conditions.

**Table 1:** Clinical data

	Total	Age <21 years	Age 21–50 years	Age >50 years
Sex ratio (m:f)	17:22	5:5	5:13	6:4
Number patients	39	10	18	11
Secondary malrotation^a^	3	1	2	0
Symptoms at diagnosis				
Abdominal pain	31 (79 %)	7 (70 %)	16 (89 %)	8 (73 %)
Intestinal obstruction	5 (13 %)	3 (30 %)	1 (6 %)	1 (9 %)
Incidental diagnosis	3 (8 %)	0 (0 %)	2 (11 %)	1 (9 %)
Duration of symptoms				
Hours/days	3 (8 %)	1 (10 %)	1 (6 %)	1 (9 %)
Months	7 (18 %)	1 (10 %)	1 (6 %)	5 (45 %)
Years	26 (67 %)	8 (80 %)	13 (72 %)	5 (45 %)
During childhood	19 (49 %)	6 (60 %)	10 (56 %)	3 (27 %)
Imaging^b^				
UGI	4	2	0	2
CT	32	7	16	9
MRI	1	0	1	0
“Whirlpool sign”^c^	7/33 (21 %)	1/5 (20 %)	3/13 (23 %)	3/7 (43 %)
Treatment				
Conservative treatment	8 (21 %)	0 (0 %)	4 (22 %)	4 (36 %)
Ladd’s surg. procedure	31 (79 %)	10 (100 %)	14 (78 %)	7 (64 %)
Midgut volvulus without impaired bloodflow	7	1	5	1
Midgut volvulus with impaired blood flow	8	5	1	2
Resection small intestine	4	3	0	1
Recurrencies	5 (16 %)	2 (20 %)	2 (14 %)	2 (29 %)

^a^CDH n = 1, gastroschisis n = 1, omphalocele n = 1

^b^“Imaging” denotes the radiologic procedure that lead to diagnosis. Two patients had no imaging due to emergency surgery (Age ≤20 years n = 1, age 21–50 years n = 1)

^c^Out of 33 patients where CT-studies were available for reviewing

**Table 2:** Preoperative symptoms from medical charts (n = 31) and postoperative symptoms from a telephone interview (n = 26)

	Total	Age <21 years	Age 21–50 years	Age >50 years
Symptoms preop (one or more symptoms from medical charts, n = 31)				
Number	31	10	14	7
Fullness after meals	25 (81 %)	7 (70 %)	14 (100 %)	4 (57 %)
Pain	29 (94 %)	9 (90 %)	13 (93 %)	7 (100 %)
Constipation	13 (42 %)	4 (40 %)	7 (50 %)	2 (29 %)
Symptoms postoperative (from a telephone interview, n = 26)				
Number	26	5	14	7
Free of symptoms	10 (38 %)	2 (40 %)	4 (29 %)	4 (57 %)
Fullness after meals	8 (31 %)	1 (20 %)	5 (36 %)	2 (29 %)
Pain (chronic)	12 (46 %)	3 (60 %)	6 (43 %)	3 (43 %)
Pain (“malrotation-like”)	1 (4 %)	1 (20 %)	0 (0 %)	0 (0 %)
Constipation	8 (31 %)	0 (0 %)	8 (57 %)	0 (0 %)
Symptoms postoperative	16 (62 %)	3 (60 %)	10 (71 %)	3 (43 %)
Improved QoL	25 (96 %)	4 (80 %)	14 (100 %)	7 (100 %)

Concomitant malformations were observed in 15 patients (38 %), including seven patients with CNS disturbances and mental retardation. Other malformations noticed were bicorn uterus, vaginal atresia, double ureters, Tuberose sclerosis, Mb Hirschprung, pelvic kidney, Cornelia de Lange syndrome and scoliosis. Eight patients had a history of disease within the hepatobiliary and pancreatic system with a history of pancreas divisum and in four cases pancreatitis. Six further patients had gastrointestinal motility disturbances, verified by small bowel manometry and/or full thickness specimens.

### Radiological findings

**Fig. 1:**
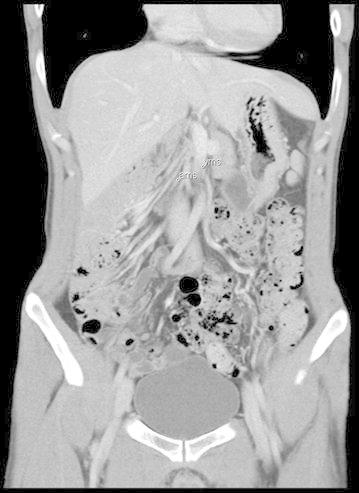
CT scan showing inverted vessels, front view

**Fig. 2:**
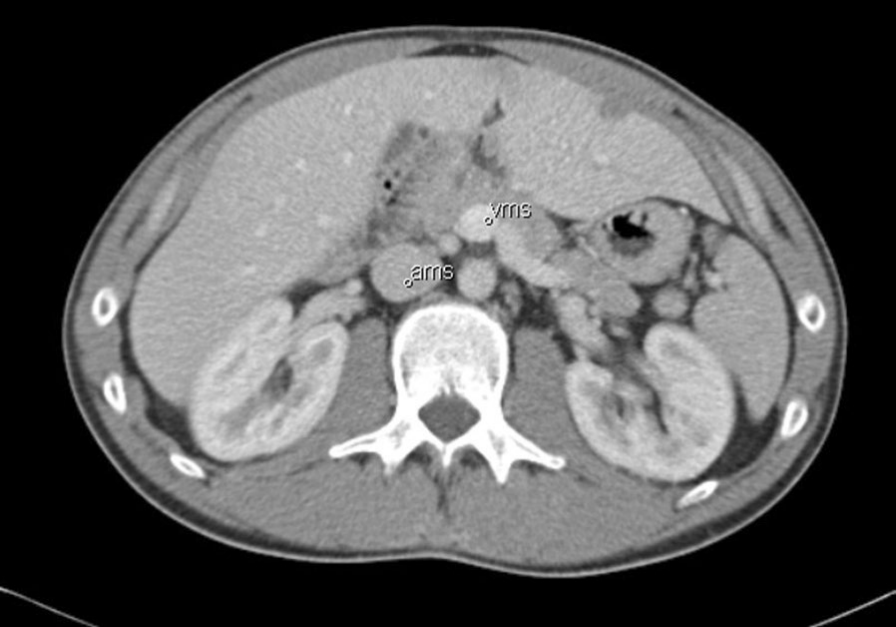
CT scan in axial position showing the inverted vessels

**Fig. 3:**
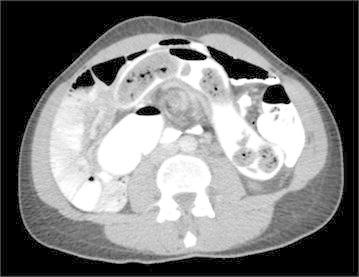
Whirl pool sign where when the mesenteric of the small intestine has been twisted

Investigation with multi-detector computer tomography was used in 32 cases and MRI in one. The three cardinal radiological diagnostic criteria were identified in 19 of the cases. In all but one of the patients, the small bowel was located to the right with a pathological vertical course of the duodenum that failed to traverse the vertebral spine. In the remaining patient, the duodenal course initially crossed the midline to the left, but turned back again forming a loop. In addition, the ascending colon had a short attachment to the parietal left side. In 22 cases the caecum had the expected abnormal position according to radiology, whereas the caecum in the remaining 11 patients was located on the right side. It was later revealed during surgery that in all these cases the ascending colon was mobile and not fixed to the parietal abdominal wall, except in one case where the right flexure of colon was shortly attached. Malposition of the superior mesenteric artery and vein was noted radiologically in 26 patients, of whom 25 had inverted vessels and one presented vessels in a vertical position (Figs. 1, 2). A “whirlpool-sign” signifying a presence of rotation of the bowel could be detected in seven cases (Fig. 3).

### Surgery

**Fig. 4:**
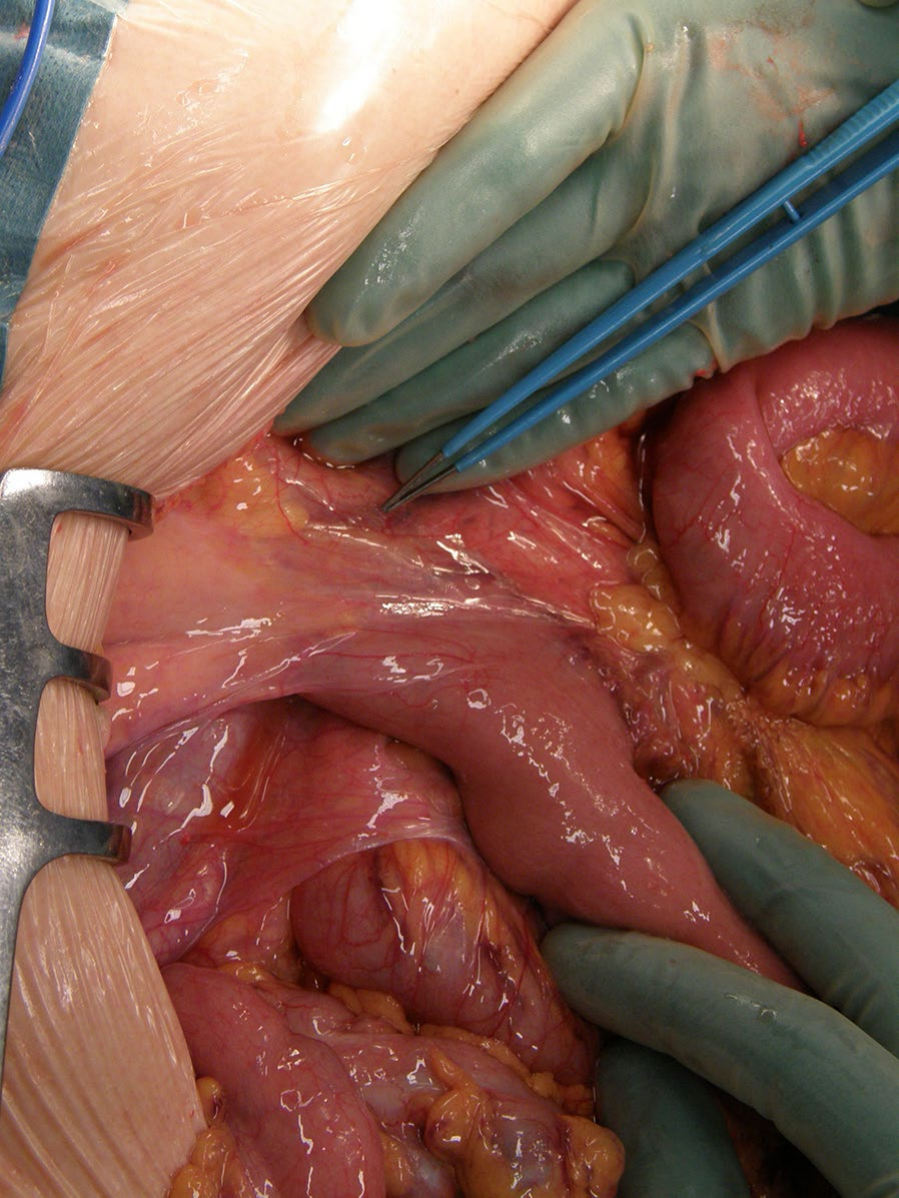
Ladds band to be dissected and removed

Thirty-one patients were operated (Fig. 4). Sixteen patients had undergone previous abdominal surgery before the Ladd procedure, with chart notifications of intestinal malrotation in 11 of the cases. Emergency surgery was performed in 9 of 31 cases. In three patients the operation was performed semi-urgently because of progression of abdominal complaints. One patient had a complete mid-gut volvulus causing ischemia and necrosis of the bowel, necessitating resection of the entire small bowel. Another seven patients exhibited signs of acute volvulus, compromising circulation in a segment of the small bowel (2 of them >50 years). Three of these required minor resections. There was a tendency towards an increased risk for volvulus in the younger patients (Table 1).

**Fig. 5:**
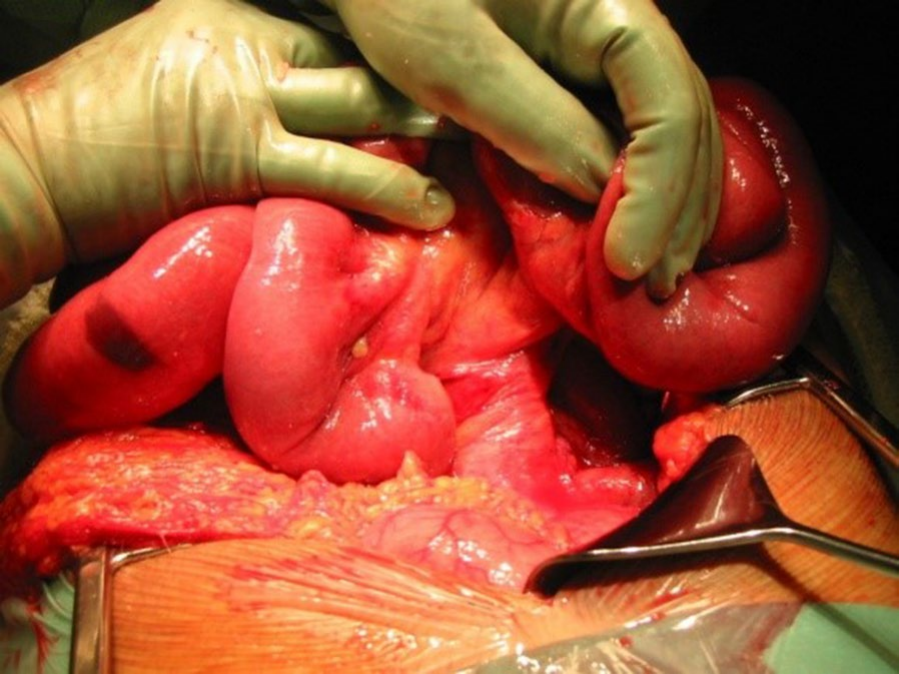
Twisting of the small bowel

Seventeen symptomatic patients were operated on electively after a radiological diagnosis. Appendectomy was performed in all cases where the appendix still remained, in order to avoid future diagnostic problems caused by the new position of the intestines in the abdomen. In 27 patients twisting of the mesentery between 1 and 3 turns was described at the initiation of surgery (Fig. 5).

Eight patients chose a conservative attitude awaiting eventual more disabling symptoms, an attitude that was more common in the older patients.

### Postoperative clinical outcome

**Fig. 6:**
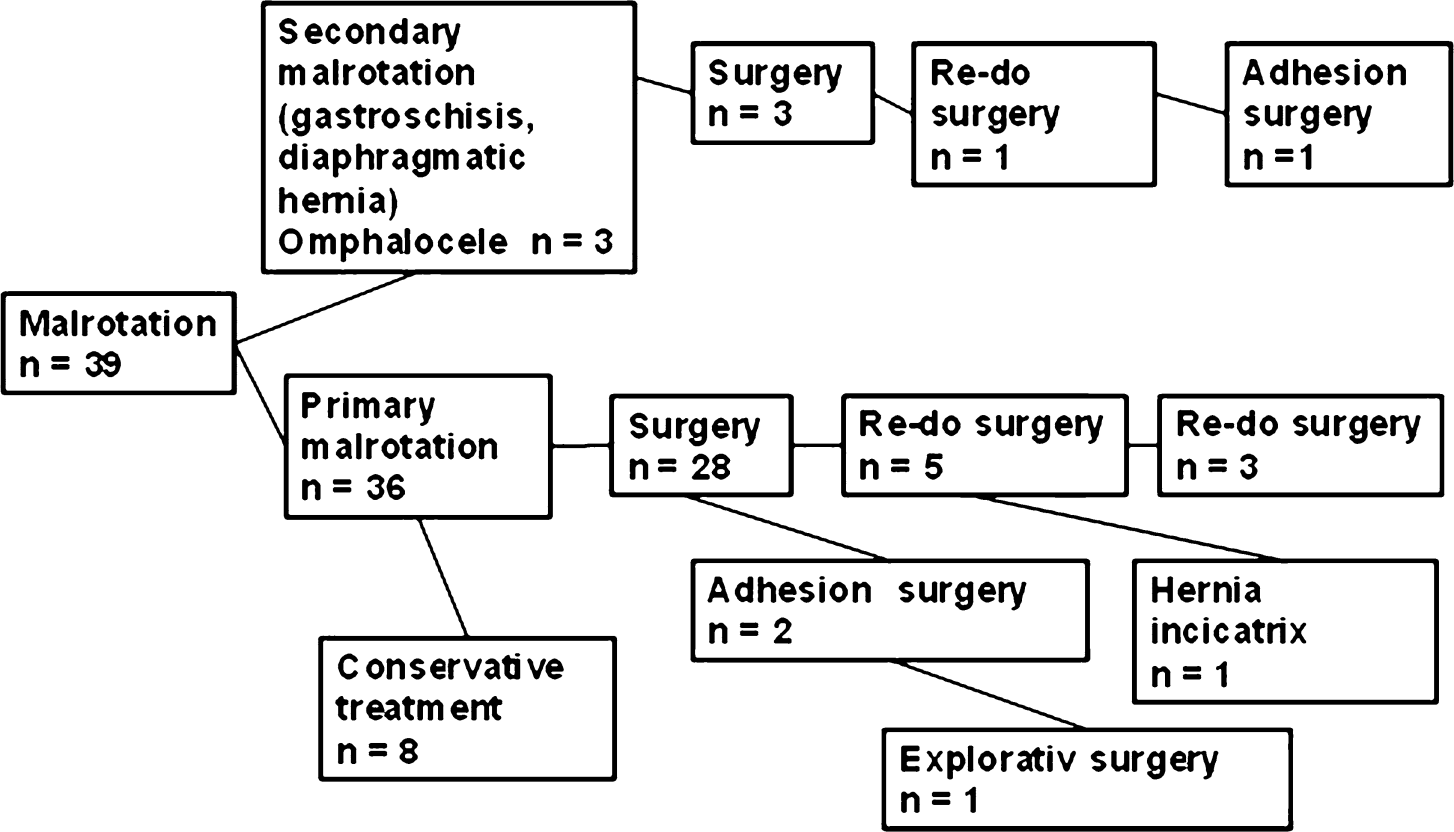
Flowchart over the procedure for the patients

Twenty-seven patients had an uneventful postoperative course, leaving the hospital within a week. Three patients had a prolonged hospital stay due to transient postoperative intestinal failure and one died shortly postoperatively in the aftermath of midgut volvulus with total bowel necrosis. An early routine follow-up after 6 weeks confirmed that all patients except one were relieved from episodes of intense abdominal pain. Caretakers of the mentally disabled patients stated that their patients exhibited less signs of distress from episodes of pain. Clinical and/or radiological signs of late recurrence appeared in six patients requiring surgery once or twice. Surgical procedures are described in a flowchart (Fig. 6).

### Mortality

Altogether five patients operated for congenital malrotation have died through the course of this study. Four of them died due to co-morbidity not related to the malrotation syndrome or surgery. These patients had all undergone a follow-up CT without signs of recurrence.

### Telephone interview

Twenty-six patients were available for a telephone interview and were asked about their situation after surgery. Details are shown in Table 2.

## Discussion

Intestinal congenital malrotation should be recognized as a reason for abdominal pain also in adults which has also been emphasized in a recent population based study by Coe et al. (2015). We describe a substantial number of symptomatic patients being diagnosed in mature age often after several years of suffering. Malrotation may present with alarming symptoms, causing life-threatening conditions which in one case resulted in death due to short bowel syndrome. We also show that young adults have a tendency towards more severe symptoms requiring emergency treatment. No statistical comparison has been made with patients suffering adhesive bowel obstruction from other reasons, and thus age and concurrent developmental disorders are the only markers identified necessitating increased awareness when considering malrotation as cause for obstruction with severe symptoms.

In this 12-year clinical study, the majority of the patients experienced a considerable improvement in their general status after surgical intervention. Nehra presents an excellent retrospective study, which includes 130 patients of all ages treated at a single institution (Nehra and Goldstein 2011). Only 30 % of the patients were below 1 year of age, and as many as 48 % were above 18 years of age at the time of diagnosis. They described a decreased risk for volvulus with age, which also was confirmed among adult cases in the present study. Consequently, a conservative attitude towards surgery is more reasonable in the older age group.

The increased recognition of intestinal malrotation in adults may be explained by the more frequent use of abdominal CT-scan and refinements of methods that more correctly visualize variations in the abdominal anatomy (Pickhardt and Bhalla 2002; Emanuva et al. 2011). A multi-detector CT-scan provides the possibility of following the exact course of the duodenum as well as the position of the small bowel and the caecum. Importantly, the orientation of the superior mesenteric vessels also becomes assessable, sometimes with an additional depicted rotation of the mesentery of the bowel forming a “whirlpool-sign”. This may indicate a precarious circulation of the bowel, possibly requiring rapid surgical intervention. In the present study there were 11 patients with the ascending colon located at an allured right abdominal quadrant. This confirms that a “normal-looking” anatomical finding of the colon should not rule out the malrotation diagnosis as earlier reported by El-Gohary who describes reciprocal findings in 20 % of the cases (El-Gohary et al. 2010). The entire set of radiological criteria was demonstrated in only 18 of the investigated patients. However, all patients exhibited at least one of the radiological criteria for malrotation, implying that a radiological investigation focusing on appropriate radiological signs provides at least a suspected diagnosis, while waiting further assessment. In children a contrast enema of the stomach and small intestine is usually enough to diagnose malrotation where the displacement of duodenum is clearly shown. In adults, where other reasons for intestinal obstruction are more frequent, a more detailed imaging including exact criteria prior to surgery is valuable.

One third of the patients were operated as emergencies, compared to the higher incidence of 75 % reported in pediatric series (El-Gohary et al. 2010). Many patients had ongoing abdominal discomforts since childhood, while others encountered a relatively sudden onset of symptoms leading to chronic episodes of abdominal pain. A considerable proportion of the patients in this series had reached a high age before being informed of their abnormality. Gastroenterologists and surgeons treating adults probably put less emphasis on the possibility of a congenital malformation causing the abdominal symptoms (Nehra and Goldstein 2011; Nagdeve et al. 2012).

Intestinal malrotation may have a “syndromal” appearance and is often accompanied by other anomalies (30–80 %), including developmental disorders of the CNS (Penco et al. 2007; Nagdeve et al. 2012). It is important to have a vigilant strategy for malrotation when investigating abdominal complaints in mentally disabled patients who lack the possibility to describe their symptoms. The comorbidity caused by these concurrent disorders may be one reason for the high mortality during follow up (5/39), since only one patient died from complications after surgery in terms of short bowel after resection of ischemic intestine.

In pediatric reports, the recurrence rate after Ladd’s procedure is considered low with a reported incidence between 2 and 7 % (El-Gohary et al. 2010; Freitz and Vos 1997). The higher recurrence rate reported here is partly explained by a learning curve among the involved surgeons, but more long-lasting preoperative symptoms may also add to the complexity of surgical problems. Interestingly, it has been shown that also children operated later during childhood has a higher incidence of reoperation (Durkin et al. 2008). Chronic inflammatory changes in the intestinal wall may have affected the outcome of the Ladd’s procedure and may influence on postoperative pain.

Seven patients with a radiological malrotation diagnosis have not yet undergone surgery, claiming that they currently experience only mild symptoms and wish for a conservative approach. Today, many authors advocate surgical correction of malrotation due to the difficulty in predicting who will be striked by torsion of the midgut, bringing an urgent, life-threatening condition in the future. Furthermore, we cannot be certain that patients without complaints are truly free from symptoms (Raitio et al. 2015; Moldrem et al. 2008).

## Conclusion

Intestinal malrotation shall be regarded as a malformation affecting all age groups since it is obviously more common in the adult population than earlier anticipated. A properly performed contrast enhanced computer tomography reveals the malformation and enables surgical treatment and relieve of symptom also in adults with a history of long-periods of abdominal complaints. In addition, and most importantly, acute obstruction with volvulus occurs in all ages and needs emergency surgery.
